# Efficacy and safety of tolvaptan versus placebo in the treatment of patients with autosomal dominant polycystic kidney disease: a meta-analysis

**DOI:** 10.1007/s11255-022-03353-8

**Published:** 2022-09-07

**Authors:** Jingkui Lu, Wei Xu, Lifeng Gong, Min Xu, Weigang Tang, Wei Jiang, Fengyan Xie, Liping Ding, Xiaoli Qian

**Affiliations:** 1 Department of Nephrology, Wujin Hospital Affiliated With Jiangsu University, No. 2 Yongning Road, Changzhou city, 213000 Jiangsu Province China; 2 Department of Nephrology, The Wujin Clinical College of Xuzhou Medical University, No. 2 Yongning Road, Changzhou City, 213000 Jiangsu Province China

**Keywords:** Tolvaptan, Placebo, Autosomal dominant polycystic kidney disease, Meta-analysis

## Abstract

**Objective:**

The objective of this meta-analysis was to compare the efficacy and drug safety of tolvaptan with placebo for autosomal dominant polycystic kidney disease (ADPKD).

**Methods:**

The PubMed, Embase, and Cochrane Library databases were searched from inception to September 10, 2021. Eligible studies comparing tolvaptan and placebo in the treatment of patients with ADPKD were included. Data were analysed using Review Manager Version 5.3.

**Results:**

Thirteen studies involving 3575 patients were included in the meta-analysis. Compared with placebo, tolvaptan had a better effect on delaying eGFR decline (MD 1.27, 95% CI 1.24–1.29, *P* < 0.01) and TKV increase (MD − 3.01, 95% CI − 3.55 to − 2.47, *P* < 0.01) in ADPKD treatment. Additionally, tolvaptan reduced the incidence of complications such as renal pain (OR 0.71, 95% CI 0.58–0.87, *P* < 0.01), urinary tract infection (OR 0.69, 95% CI 0.54–0.89, *P* < 0.01), haematuria (OR 0.68, 95% CI 0.51–0.89, *P* < 0.01), and hypertension (OR 0.66, 95% CI 0.52–0.82, *P* < 0.01). However, tolvaptan was associated with a higher incidence rate of adverse events such as thirst (OR 8.48 95% CI 4.53–15.87, *P* < 0.01), polyuria (OR 4.71, 95% CI 2.17–10.24, *P* < 0.01), and hepatic injury (OR 4.56, 95% CI 2.51–8.29, *P* < 0.01).

**Conclusion:**

Tolvaptan can delay eGFR decline and TKV increase and reduce complications such as renal pain, urinary tract infection, haematuria, and hypertension in the treatment of ADPKD. However, tolvaptan increases the adverse effects of thirst, polyuria and hepatic injury.

## Introduction

Autosomal dominant polycystic kidney disease (ADPKD) is a single-gene disease and the most common inherited progressive kidney disease, characterized by the progressive development of bilateral kidney cysts and variable progression to end-stage kidney disease renal disease (ESRD) [1, 2]. According to statistics, ADPKD is the fourth leading cause of end-stage kidney disease (ESRD) in adults [3–6]. The cause of ADPKD is related to mutations in two major genes, PKD1 and PKD2, and the rare genes, GANAB and DNAJB11 [1]. Currently, the treatment for ADPKD is limited to the management of symptoms and complications [7]. In recent years, tolvaptan, a vasopressin V2-receptor antagonist, was found to inhibit adenosine-3′,5′-cyclic monophosphate (cAMP) production and limit kidney cyst development and growth [8, 9]. Some studies have compared tolvaptan with placebo for ADPKD concerning efficacy and safety, and the results were controversial. Our meta-analysis was conducted to compare the efficacy and drug safety of tolvaptan and placebo treatment for ADPKD patients to provide a useful reference for clinicians.

## Materials and methods

### Search strategy

Our meta-analysis was conducted in accordance with the Preferred Reporting Items for Systematic Reviews and Meta-Analyses and Assessing the methodological quality of systematic review guidelines. We searched the PubMed, Embase, and Cochrane Library databases from inception to September 10, 2021. The combined text and MeSH terms included autosomal dominant polycystic kidney disease, tolvaptan, and placebo. In addition, the reference lists of the included papers were manually searched to identify eligible studies. There were no language restrictions.

### Inclusion and exclusion criteria

The inclusion criteria were as follows: (i) randomized controlled trials (RCTs), cohort or case–control studies; ii) studies of patients with ADPKD; (iii) studies designed to compare tolvaptan with placebo; and (iV) primary end points of this review were recorded, including total kidney volume (TKV) and estimated glomerular filtration rate (eGFR), and the secondary end points were reported, including the incidence rates of renal pain, urinary tract infection, haematuria, and hypertension. Adverse events such as thirst, polyuria, and hepatic injury were also examined.

The exclusion criteria were as follows: (i) case series, comments, and reviews; (ii) lack of relevant outcome data; (iii) age < 18 years, eGFR < 15 ml/min per 1.73 m^2^, anticipation of renal replacement therapy, systolic blood pressure < 90 mmHg, and serious cardiac or hepatic disease.

### Data extraction and quality assessment

Data were extracted independently by two investigators using standard data extraction forms. In the case of disagreement, a third investigator was consulted. We extracted the following data: first author, year of publication, location, study design, follow-up period, age, sex, sample size, and outcomes. The Cochrane assessment tool was used to evaluate the quality of RCTs [10]. The Newcastle–Ottawa scale (NOS) was used to evaluate the quality of nonrandomized studies [11].

### Statistical analysis

This meta-analysis was performed using Review Manager Version 5.3 (Cochrane Collaboration). We summarized treatment outcomes as odds ratios (ORs) for categorical variables and weighted mean differences for continuous variables with 95% confidence intervals (CIs). *P* < 0.05 was considered statistically significant. We used the *I*^2^ statistic to assess heterogeneity among studies. We considered *I*^2^ > 50% and *P* < 0.10 to indicate significant heterogeneity. Meta-analysis with insignificant heterogeneity was performed using the fixed effects model. For meta-analyses with significant heterogeneity, the random effects model was used. Publication bias was assessed using subgroup analysis or sensitivity analysis.

## Results

### Study selection and characteristics

**Fig. 1:**
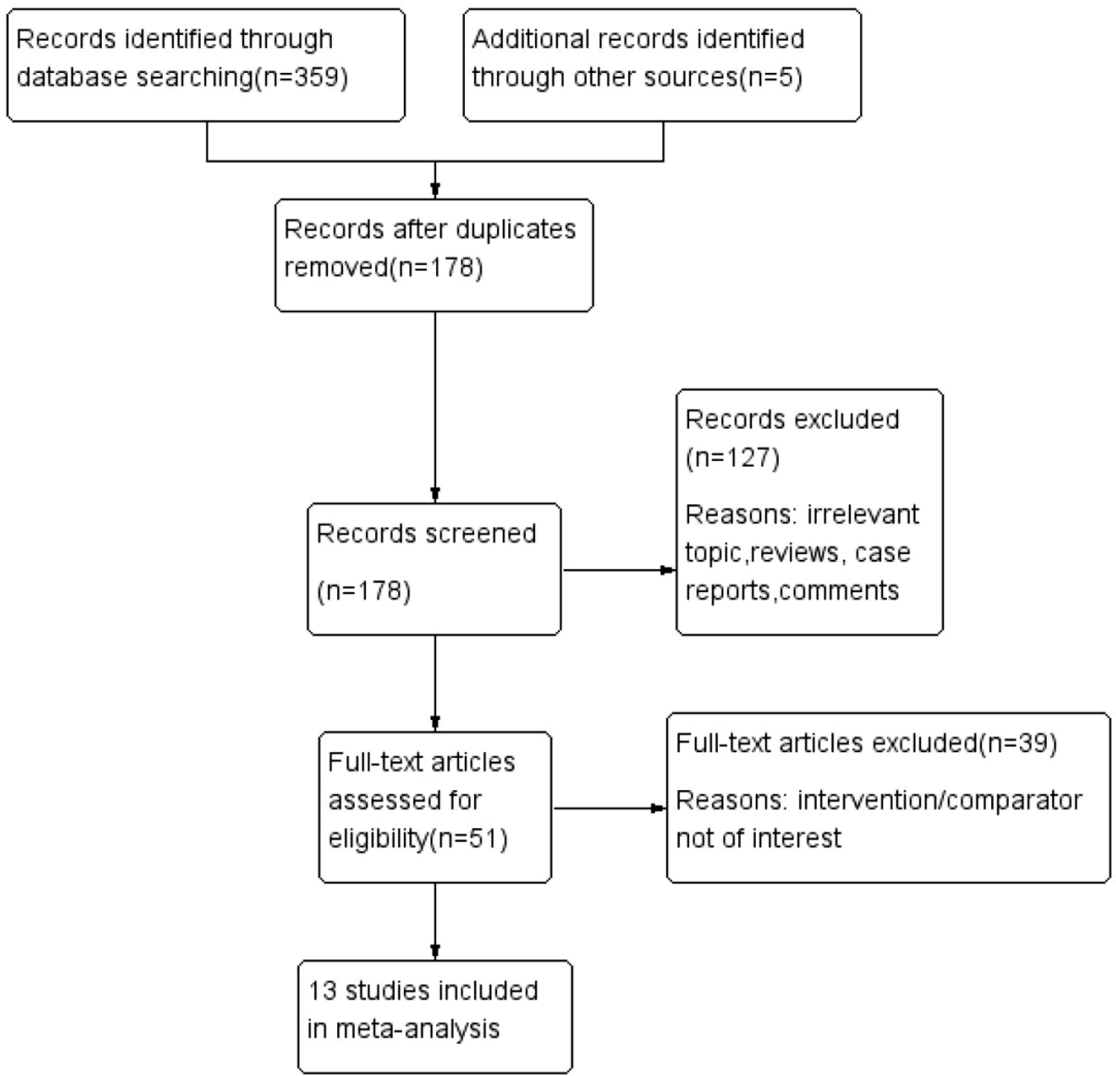
Flow diagram of the literature search

A flow diagram of the selection process is shown in Fig. 1. Finally, 13 studies were included in this analysis [12–24]. Overall, 2011 patients were included in the tolvaptan group, and 1564 patients were included in the placebo group. The follow-up period of the Edwards study was over 5 years, and the follow-up periods of other studies ranged from 2 to 36 months. The data of six studies were extracted from the TEMPO trial of Torres [14, 15, 17, 22–24]. In addition, Torres conducted the REPRISE trial in 2017. The risk of bias in the included RCTs was moderate. The nonrandomized studies achieved scores of ≥ 6 points, which indicated high quality. The baseline characteristics of these studies are listed in Table 1. The Cochrane assessment is listed in Table 2, and the NOS assessment is listed in Table 3.

**Table 1:** Characteristics of the included studies

Study (year)	Country	Study design	Follow-up (year)	Sample size	Mean age (year)	Male (%)	eGFR (ml/min)	TKV increasing ratio (%/year)
Edwards et al. (2018) [12]	America	Paired design	4.6 ± 2.8 6.9 ± 3.6	Tolvaptan:97 Placebo:97	44 ± 10 44 ± 9	37 37	64 ± 25 64 ± 25	–
Lai et al. (2020) [13]	Italy	Prospective study	1	Tolvaptan:10 Placebo: 26	42.5 ± 7.0 36.7 ± 9.1	70 57.7	56 ± 15 61 ± 20	–
Casteleijn et al. (2016) [14]	129 sites worldwide	RCT	1.5	Tolvaptan:961 Placebo:484	39 ± 7 39 ± 7	51.5 51.9	81.4 ± 21.0 82.1 ± 22.7	1705 ± 921 1668 ± 873
Torres et al. (2016) [15]	129 sites worldwide	RCT	1.5	Tolvaptan:958 P lacebo:481	39 ± 7	52	81 ± 22	1692
Torres et al. (2017) [16]	213 sites worldwide	RCT	1	Tolvaptan:683 Placebo:687	47.3 ± 8.2 47.2 ± 8.2	50.8 48.5	40.7 ± 10.9 41.4 ± 11.2	–
Torres et al. (2012) [17]	129 sites worldwide	RCT	1.5	Tolvaptan:961 Placebo:484	39 ± 7 39 ± 7	51.5 51.9	81.4 ± 21.0 82.1 ± 22.7	1705 ± 921 1668 ± 873
Yamamoto et al. (2019) [18]	Japan	Paired design	2.8 ± 0.9	Tolvaptan:41 Placebo:41	–	–	50.0 ± 19.6 54.0 ± 27.7	1172 ± 607 1028 ± 775
Higashihara et al. (2011) [19]	America and Japan	RCT	3	Tolvaptan:51 Placebo:102	–	33.3 33.3	62 ± 20.1 62 ± 19.1	1635 ± 978 1422 ± 725
Kai et al. (2018) [20]	Japan	Prospective study	1	Tolvaptan:34 Placebo:84	48.5 ± 12.0	53	56.0 ± 30.2	1814 ± 1390
Perrone et al. (2020) [21]	Multicenter	RCT	1/6	Tolvaptan:134 Placebo:43	34.0 33.9	45.5 53.5	85.4 85.1	1674.9 1728.8
Heida et al. (2021) [22]	129 sites worldwide	RCT	1.5	Tolvaptan:961 Placebo:484	39 ± 7 39 ± 7	51.5 51.9	81.4 ± 21.0 82.1 ± 22.7	1705 ± 921 1668 ± 873
Raina et al. (2020) [23]	129 sites worldwide	RCT	1.5	Tolvaptan:39 Placebo:24	39 ± 7 39 ± 7	44 50	110.5 ± 15.6 120.0 ± 19.9	634 753
Muto et al. (2015) [24]	Japan	RCT	1.5	Tolvaptan:118 Placebo:59	38.7 ± 6.1 40.4 ± 5.6	50 59.3	72.74 ± 15.82 70.16 ± 16.19	1456 ± 559.2 1567 ± 638.3

**Table 2:** Quality assessment of randomized control trial

Study	Random sequence generation	Allocation concealment	Blinding of participants and personnel	Incomplete outcome data	Selective reporting	Other bias
Casteleijn et al. (2016) [14]	+	+	+	−	−	+
Torres et al. (2016) [15]	+	+	+	+	+	+
Torres et al. (2017) [16]	+	+	+	−	−	+
Torres et al. (2012) [17]	+	+	+	+	+	+
Perrone et al. (2020) [21]	+	+	+	+	+	?
Heida et al. (2021) [22]	+	+	+	+	+	?
Raina et al. (2020) [23]	+	+	+	+	+	?
Higashihara et al. (2011) [19	+	+	+	−	−	+
Muto et al. (2015) [24]	+	+	+	+	+	+

The randomized control trial was evaluated using the Cochrane assessment tool

+  low risk of bias, ? unclear risk of bias, – high risk of bias

**Table 3:** Quality assessment of nonrandomized control trial

Studies	Selection	Comparability	Outcome	Score
Edwards et al. (2018) [12]	**★★★★**	★	**★★★**	8
Lai et al. (2020) [13]	**★★★★**	**★**	**★★★**	8
Yamamoto et al. (2019) [18]	**★★★**	**★**	**★★**	6
Kai et al. (2018) [20]	**★★★**	**★**	**★★**	6

The Cohort studies were evaluated using the Newcastle–Ottawa scale, which comprised the study of selection (representativeness of the exposed group, representativeness of the nonexposed group, ascertainment of exposure, demonstration that outcome of interest was not present at start of study), group comparability (controls for the most important factor, controls for any additional factor), outcome measures (assessment of outcome, was follow-up long enough for outcomes to occur, adequacy of follow-up of cohorts), a total of nine points. **★**, 1 point

### Meta-analysis results

#### Primary end points

**Fig. 2:**
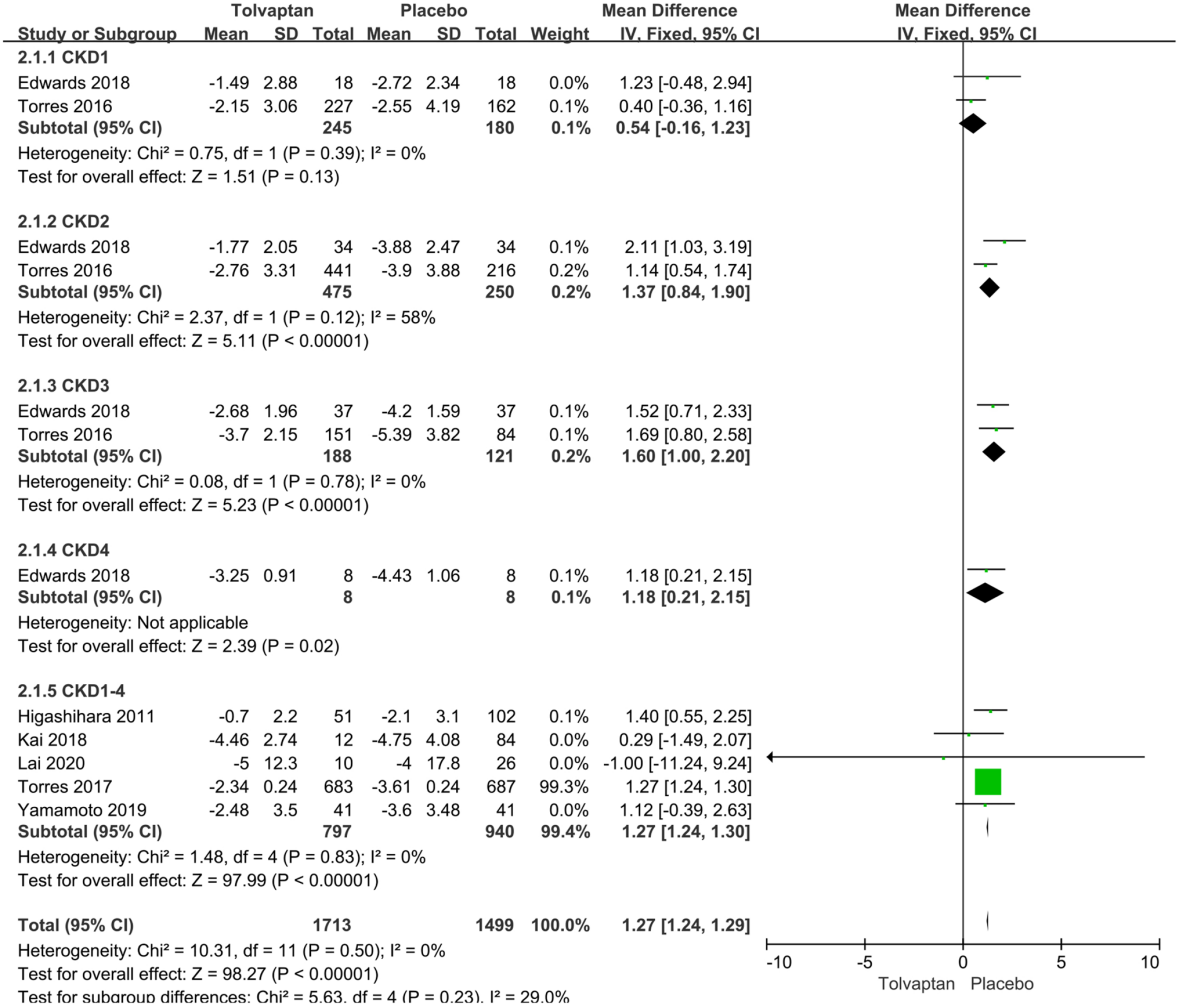
Forest plot of eGFR slope (ml/min per 1.73 m^2^ per year) between tolvaptan and placebo

*eGFR* Data about eGFR slope were reported in seven articles. Subgroup analysis revealed that there was no significant difference between tolvaptan and placebo on the eGFR slope at chronic kidney disease (CKD) stage 1 (MD 0.54, 95% CI − 0.16 to 1.23, *P* = 0.13). The eGFR slope in the tolvaptan-treated patients was lower than that in the placebo-treated patients at CKD stages 2, 3 and 4 (MD 1.37, 95% CI 0.84–1.90, *P* < 0.01) (MD 1.60, 95% CI 1.00–2.20, *P* < 0.01) (MD 1.18, 95% CI 0.21–2.15, *P* = 0.02). Overall, tolvaptan had a better effect on the rate of eGFR decline (MD 1.27, 95% CI 1.24–1.29, *P* < 0.01) (Fig. 2).

**Fig. 3:**
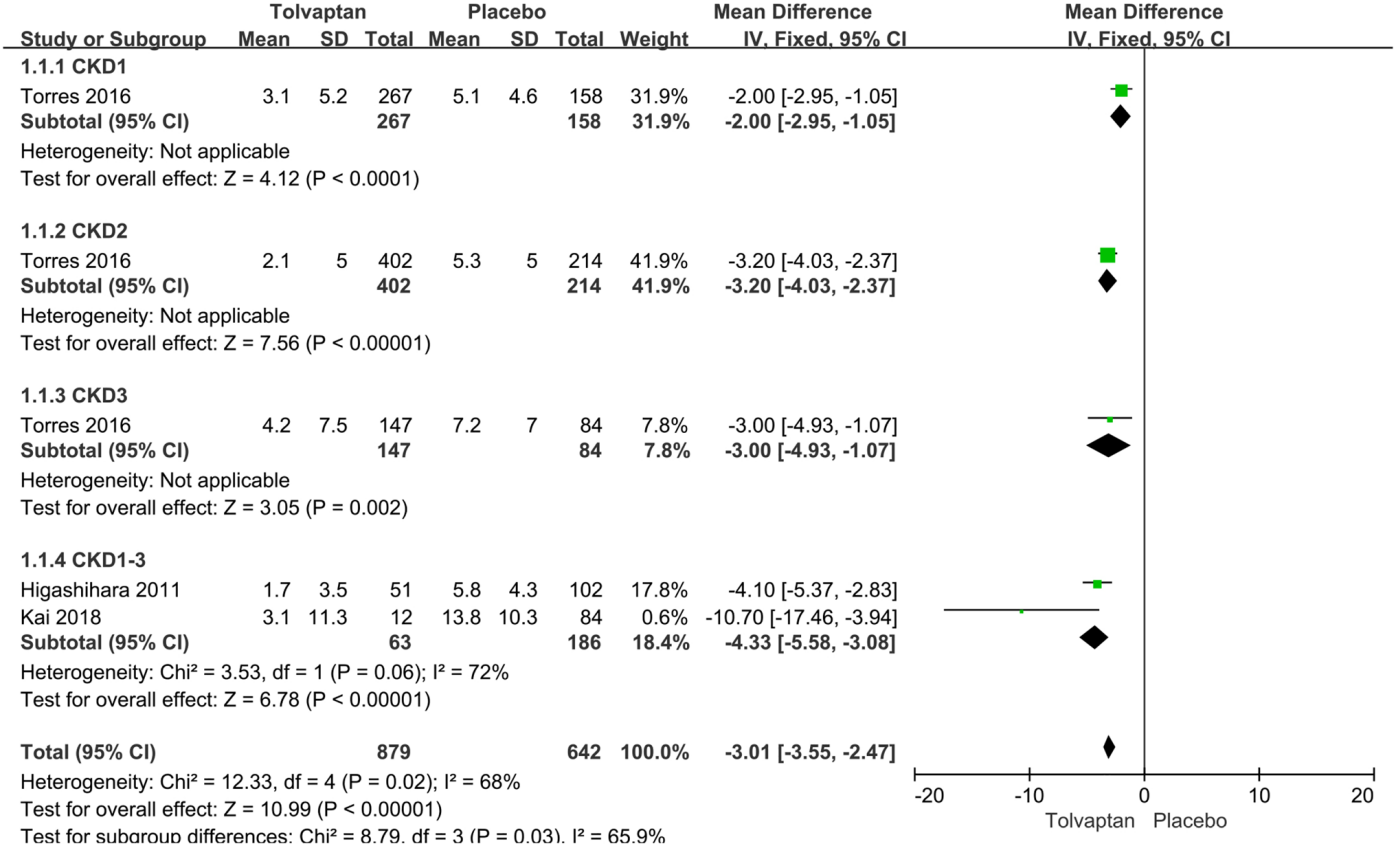
Forest plot of TKV increase rate (%/year) between tolvaptan and placebo

*TKV* Data about the annual rate of change in the TKV were reported in four articles. Subgroup analysis found that the increases in TKV in the tolvaptan-treated patients were lower than those in the placebo-treated patients at CKD stages 1, 2 and 3 (MD − 2.00, 95% CI − 2.95 to − 1.05, *P* < 0.01) (MD − 3.20, 95% CI − 4.03 to − 2.37, *P* < 0.01) (MD − 3.00, 95% CI − 4.93 to − 1.07, *P* < 0.01). As a whole, tolvaptan had a better effect on the annual rate of change in the TKV (MD -3.01, 95% CI − 3.55 to − 2.47, *P* < 0.01) (Fig. 3).

#### Secondary end points

**Fig. 4:**
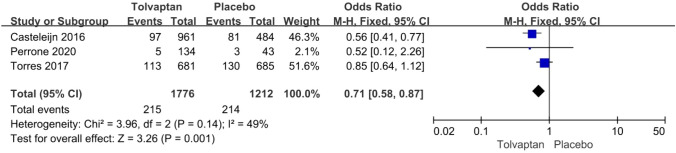
Forest plot of renal pain between tolvaptan and placebo

*Renal pain* The incidence rate of renal pain was reported in three articles: 215/1776 (12.1%) for the tolvaptan group and 214/1212 (17.7%) for the placebo group. Tolvaptan-treated patients had a lower incidence rate of renal pain (OR 0.71, 95% CI 0.58–0.87, *P* < 0.01) (Fig. 4).

**Fig. 5:**
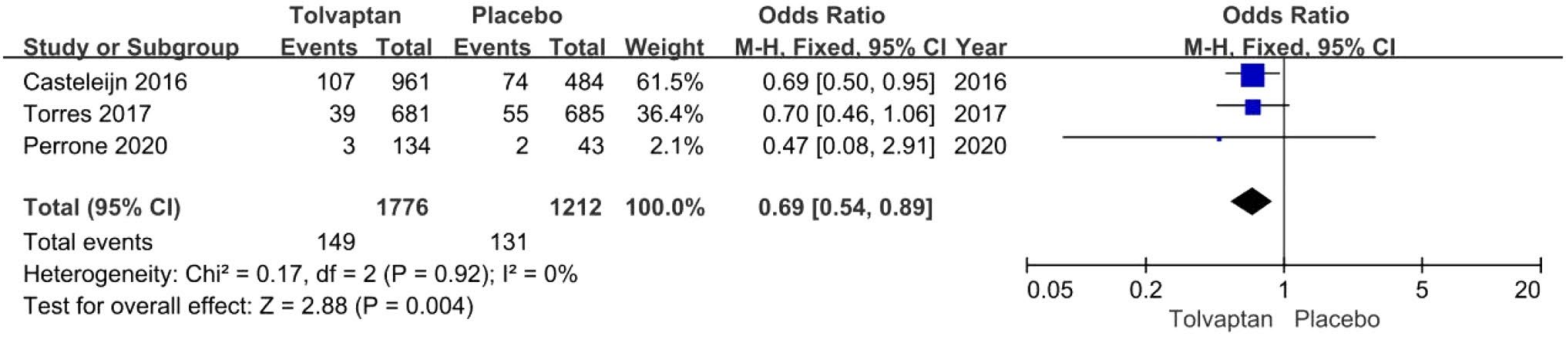
Forest plot of urinary tract infection between tolvaptan and placebo

*Urinary tract infection* The incidence rate of urinary tract infection was reported in three articles: 149/1776 (8.4%) for the tolvaptan group and 131/1212 (10.8%) for the placebo group. Tolvaptan-treated patients had a lower incidence rate of urinary tract infection (OR 0.69, 95% CI 0.54–0.89, *P* < 0.01) (Fig. 5).

**Fig. 6:**
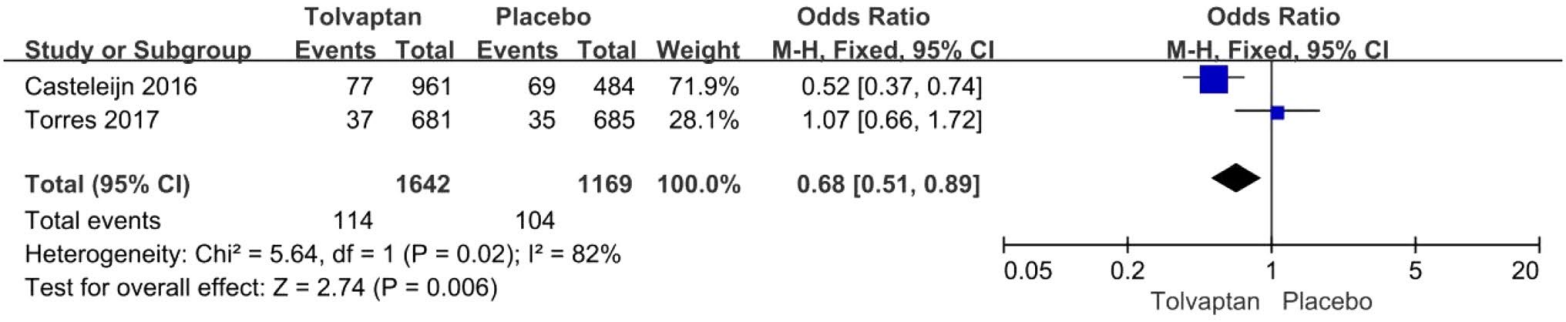
Forest plot of haematuria between tolvaptan and placebo

*Haematuria* The incidence rate of haematuria was reported in two articles: 114/1642 (6.9%) for the tolvaptan group and 104/1169 (8.9%) for the placebo group. Tolvaptan-treated patients had a lower incidence rate of haematuria (OR 0.68, 95% CI 0.51–0.89, *P* < 0.01) (Fig. 6).

**Fig. 7:**
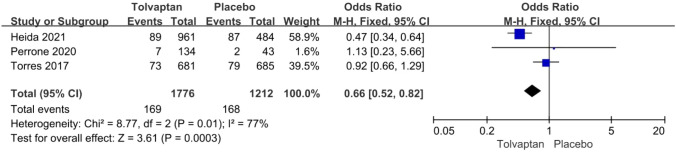
Forest plot of hypertension between tolvaptan and placebo

*Hypertension* The incidence rate of hypertension was reported in three articles: 169/1776 (9.5%) for the tolvaptan group and 168/1212 (13.8%) for the placebo group. Tolvaptan-treated patients had a lower incidence rate of hypertension (OR 0.66, 95% CI 0.52–0.82, *P* < 0.01) (Fig. 7).

#### Adverse events of thirst, polyuria, hepatic injury

**Fig. 8:**
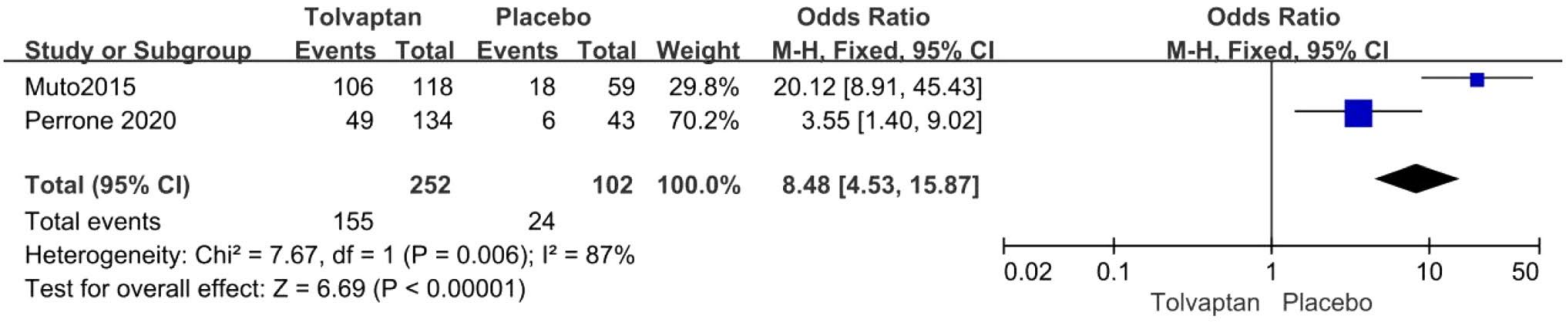
Forest plot of thirst between tolvaptan and placebo

**Fig. 9:**
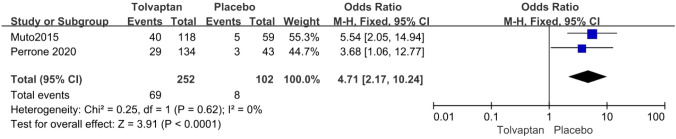
Forest plot of polyuria between tolvaptan and placebo

**Fig. 10:**
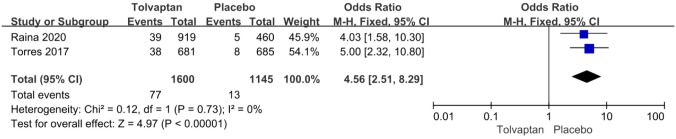
Forest plot of hepatic injury between tolvaptan and placebo

The incidence rate of common adverse events was reported in four articles. Tolvaptan-treated patients had higher incidence rates of thirst (OR 8.48 95% CI 4.53–15.87, *P* < 0.01), polyuria (OR 4.71, 95% CI 2.17–10.24, *P* < 0.01), and hepatic injury (OR 4.56, 95% CI 2.51–8.29, *P* < 0.01) (Figs. 8, 9 and 10).

### Sensitivity analyses

A sensitivity analysis of the outcomes concerning eGFR and TKV was used to judge the dependability of the results. We deleted one study at a time, and the results still showed that tolvaptan had a better effect on the rate of eGFR decline and the annual rate of change in the TKV.

## Discussion

ADPKD patients have a high risk of progressing to ESRD, which usually occurs after the age of 60 years [26]. Effective intervention for the growth of renal cysts in ADPKD patients at an early stage is of great clinical significance for delaying the progression of ESRD. Our meta-analysis revealed that tolvaptan had a better effect on delaying eGFR decline and TKV increase in ADPKD treatment. Additionally, tolvaptan reduced the incidence of complications such as renal pain, urinary tract infection, haematuria, and hypertension. However, tolvaptan has a higher incidence rate of adverse events, such as thirst, polyuria, and hepatic injury, than placebo.

The eGFR is easy to check, and it is an effective parameter for assessing the progression of ADPKD disease and the efficacy of tolvaptan therapy [7]. In our meta-analysis, as a whole, tolvaptan had a better effect on delaying eGFR decline. The mean annual decrease in eGFR among patients with CKD stage 1 treated with tolvaptan was lower than that in the placebo group, but there was no significant difference. The probable cause is that the eGFR decline was relatively slow in ADPKD patients at CKD stage 1 during a short follow-up period, so it might not show an obvious benefit of tolvaptan [15]. In addition, the European Renal Association proposed that patients aged 40–50 years with CKD stages 1 and 2 or patients 30–40 years with CKD stage are identified as slow progressors and not appropriate for tolvaptan treatment [27]. We found that the annual rate of eGFR decline gradually increased in both the tolvaptan and placebo groups as patients moved to a higher CKD stage. However, among patients at CKD stages 2, 3 and 4, the annual rates of eGFR decline in the tolvaptan group were all obviously lower than those in the placebo group, which showed that tolvaptan is effective in slowing the rate of eGFR at either the early or late stage of ADPKD. Additionally, Edwards and Torres followed up with patients for a long period of time to observe the effects of tolvaptan treatment in their study, suggesting that tolvaptan provides sustained and cumulative benefits of slowing the rate of eGFR for ADPKD patients [12, 28].

TKV growth precedes changes in GFR and directly reflects ADPKD disease progression [29]. Our meta-analysis showed that tolvaptan was similarly effective in reducing the increase rate of TKV in ADPKD patients who had CKD stage 1–3 at baseline. The effect on reducing TKV growth was due to slowing fluid secretion and decreasing cell proliferation. Additionally, the study of Torres showed that tolvaptan provides sustained benefits of reducing the increase rate of TKV during the first, second and third years [15]. Tolvaptan treatment to reduce the rate of TKV growth is accompanied by a slower rate of eGFR decline [19].

Our meta-analysis showed that tolvaptan reduced the incidence of renal pain, urinary tract infection, and haematuria. A previous study found that a large TKV was related to the occurrence of these renal complications in ADPKD, so tolvaptan reduced these complications by reducing TKV [30, 31]. Another mechanism is that tolvaptan induces polyuria, which might explain the lower incidence of urinary tract infection and kidney stones because an increase in water intake is related to a lower incidence of urinary tract infection and kidney stones in the general population. Additionally, a lower incidence of urinary tract infection and kidney stones is related to a lower incidence of renal pain and haematuria [32]. In addition, our meta-analysis showed that tolvaptan reduced the incidence of hypertension. Some studies showed that tolvaptan did not increase levels of renin or aldosterone in contrast to other diuretics [33]. However, there were influencing factors, such as the use of other antihypertensive drugs and the method of blood pressure measurement.

The administration of tolvaptan can increase the incidence of drug-related adverse effects, so clinicians should evaluate the beneficial and adverse effects when prescribing treatment regimens for ADPKD patients. Tolvaptan had the main adverse effects related to aquaresis (such as thirst and polyuria), which did not rise to a level indicating disruption of the quality of life [21]. Tolvaptan increases urine output by its mechanism of action, so the patients need to maintain good hydration, which can reduce the incidence of hypernatraemia. In addition, liver function injury is another frequent adverse event that occurs between 60 and 240 days after the start of tolvaptan treatment and becomes less frequent thereafter. More frequent monitoring of liver enzyme levels and earlier interruption of therapy probably reduced the frequency of liver function injury [16].

There were some limitations in our meta-analysis. First, in recent years, tolvaptan was approved for the treatment of rapidly progressive ADPKD in adults [7]. Age, height-adjusted TKV and eGFR were effective parameters for assessing the progression of ADPKD disease [34, 35]. The Mayo classification is a preferred tool that uses age and htTKV to identify patients at high risk for progression independent of renal function [36]. The estimated growth rates of TKV for patients with Mayo classifications C, D and E were > 3–4.5%, > 4.5–6% and > 6%, respectively, indicating rapid disease progression. However, in our meta-analysis, the subjects were specifically identified as rapidly progressive ADPKD in only two included studies completed in 2020 [13, 21]. Second, two included trials did not describe specific doses of tolvaptan. Among the other included trials, the range of tolvaptan doses was from daily morning and afternoon doses of 30 mg and 15 mg, respectively, to 90 mg and 30 mg. Adjustment of drug dosage in most included trials is according to tolerance of patients, and few trials is according to urinary osmolarity[Uosm] target ≤ 280 mOsm/kg. Some studies showed that approximately 30% of patients receiving 90/30 mg tolvaptan did not achieve a sustained Uosm < 300 mOsm/kg [19, 25]. For those achieving the target with lower tolvaptan doses, there is no evidence that it is beneficial to further lower Uosm. In contrast, it may increase adverse effects.

## Conclusions

In the treatment of ADPKD, tolvaptan can delay eGFR decline and TKV increase. Additionally, tolvaptan reduced complications of renal pain, urinary tract infection, haematuria, and hypertension. However, tolvaptan increases the adverse effects of aquaresis and hepatic injury. To further confirm this conclusion, additional large multicentre randomized controlled trials are necessary.
